# Marrying Well

**Published:** 1880-06

**Authors:** 


### MARRYING WELL.

A REAL wife is a "help-meet," an assistant suitable for her husband; a woman who adapts herself to the situation, circumstances, and position of the man who has engaged to provide her with a house and home, and to defend and protect her until she dies. It would not be just to say that no girl educated in a boarding-school ever became a good wife; but that boarding-school girls, as a class, make the worst of wives, is the impression of many a poor fellow who has had experience in that direction.

The very first care of a young man who is about to marry, should be to select a woman of vigorous health, from among those of his own religion, of his own neighborhood, and of his own grade in society. If he is of no account, he deserves nothing higher; if he is of sterling worth, he will elevate her from the hour, toward the position which he himself merits, with the happy result, that as he rises she will rise with him, become proud of him, while he will have reason to be proud of himself, and in time will carry with him that presence and that bearing which belong to the self-reliant and to those who have a consciousness of ability and moral worth.

An important advantage in marrying from among one's neighbors is, that each party knows the social "status" of the other in a manner more perfect than is otherwise possible, and thus will all impositions be avoided; for there are multitudes of persons whose inveterate aim is to impress those whom they have married with the idea of their position, their birth, and their blood, the more so as these all are questionable. The truly well-born never speak of these things voluntarily.

Within a year, a young lady of Brooklyn picked up a foreign husband at Newport; later on, she appeared at her father's door, a refugee from the intolerable treatment of her "lord" whom she had left in Italy; she was a Quakeress by education, and married out of her sphere.

In countless instances, "educated" women have made miserable wives. The fact is, in multitudes of cases, the wife is a slave, and, like any other slave, the less she knows as an intellectual being the less galling will the yoke matrimonial be, and the more likely will she be to discharge satisfactorily the material duties of a wife, which are the ordering of the household so that it shall be the haven and the heaven of the toiling husband, and the nestling, cozy refuge of the children. The truth is, the whole system of female fashionable education is an abortion and a curse. Our daughters are not trained for wives, in the true sense of the word, but for ladies, for puppets, for dolls, for playthings. Although John Bull has a high character for doing things in the right way, in respect to the girls born to him he is about as big a fool as Jonathan. In the European orphan schools and asylums of Calcutta and Madras, the children of soldiers are, with great liberality, taken to be educated, especially the daughters of soldiers and officers who have died in their country's service; but in place of being taught needle-work, cookery, reading, writing, and arithmetic, and in the domestic duties of wife and mother, they are instructed in subjects which might be expected in a London boarding-school, and hence Dr. Mouat says he has often heard steady soldiers declare that they preferred an uneducated native wife to the best of the inmates of the institutions above mentioned, because the former was gentle, quiet, obedient, fond of staying at home, careful and tender of the children, and anxious to minister to the comfort and happiness of the husband; whereas the latter was far too often a fine lady, alike regardless and ignorant of domestic duties, fond of gossip and flirtation, and altogether ill calculated to produce happiness in her husband's household. It is precisely this that is operating in New-York and Philadelphia and Boston, and other large cities, and extending even to small towns and the country, too, to diminish the number of marriages, leaving the most beautiful blossoms to be ungathered, while the bar-room, the coffee-house, and the club are more and more crowded.

